# The Feasibility and Effects of a Telehealth-Delivered Home-Based Prehabilitation Program for Cancer Patients during the Pandemic

**DOI:** 10.3390/curroncol28030207

**Published:** 2021-06-17

**Authors:** Fiona Wu, Oloruntobi Rotimi, Roberto Laza-Cagigas, Tarannum Rampal

**Affiliations:** 1 Surgery and Anesthetics Department, East Kent Hospitals University NHS Trust, Margate CT9 4AN, UK; 2 Foundation Program, Brighton and Sussex University Hospitals NHS Trust, Brighton BN2 5BE, UK; oloruntobi.rotimi@nhs.net; 3 Kent and Medway Prehab, Chatham ME4 4TR, UK; roberto.lazacagigas@nhs.net (R.L.-C.); t.rampal@nhs.net (T.R.)

**Keywords:** prehabilitation, surgical oncology, shielding, exercise oncology, cancer care, pandemic, deconditioning

## Abstract

Patients awaiting cancer treatment were classified as “vulnerable” and advised to shield to protect themselves from exposure to coronavirus during the pandemic. These measures can negatively impact patients. We sought to establish the feasibility and effects of a telehealth-delivered home-based prehabilitation program during the pandemic. Eligible patients were referred from multiple centers to a regional prehabilitation unit providing home-based prehabilitation. The enrolled patients received telehealth-delivered prehabilitation prior to surgery and/or during non-surgical cancer treatment, which included personalized training exercises, dietary advice, medical optimization therapies, and psychological support. The primary outcome was to investigate the feasibility of our program. The secondary outcome was to investigate the relationship between our program and patient-reported outcomes (PROs). The patients completed two questionnaires (the EQ-5D-3L and the FACIT-Fatigue Scale) pre- and post-intervention. A total of 182 patients were referred during the study period. Among the 139 (76%) patients that were enrolled, 100 patients completed the program, 24 patients have still to complete, and 15 have discontinued. A total of 66 patients were able to return completed questionnaires. These patients were recruited from colorectal, urology, breast, and cardiothoracic centers. The patients significantly improved their self-perceived health (*p* = 0.001), and fatigue (*p* = 0.000). Home-based prehabilitation is a feasible intervention. The PROs improved post-intervention.

## 1. Introduction

On the 11th of March 2020, the World Health Organization declared the novel coronavirus COVID-19 outbreak as a global pandemic. The UK government adopted containment measures at the height of the pandemic to prevent the National Health Service (NHS) being overwhelmed and to contain the spread of the virus [1]. “High-risk” and medically “vulnerable” patients were advised to shield (to strictly stay at home and to reduce face-to-face interactions) to protect themselves from exposure to coronavirus. Whilst these measures reduce the risk of these patients contracting COVID-19, the prohibition of close social interaction and the restriction in movement has resulted in unintended consequences on physical function and quality of life [2,3]. Physical separation can lead to feelings of social isolation and a deterioration in mental health. Home confinement can put these patients at a greater risk of physical deconditioning and immobilization.

Prior to the pandemic, we were able to provide our cancer patients with center-based, or face-to-face, prehabilitation interventions [4,5]. Prehabilitation (“prehab”) involves identifying and promoting health-optimizing behaviors to mitigate the unwanted consequences of cancer treatment, often in the pre-operative setting to improve post-operative outcomes [6,7]. The benefits of prehabilitation for cancer patients have been well documented [8]. Much of the evidence in support of prehabilitation has studied the effects of in-person supervised programs [9,10]. There has been little attention given to telehealth-delivered and/or home-based programs [11,12,13,14]. In response to the pandemic, following the UK government’s announcement of a national lockdown, we sought to use digital technologies and telecommunication methods to continue the benefits of prehabilitation and to mitigate the consequences of shielding [15,16].

The primary objective of this study was to evaluate the feasibility of adapting our pre-existing face-to-face program to a telehealth-delivered home-based format across multiple cancer treatment pathways. The secondary objective was to investigate the effects of our intervention on patient-reported outcomes, with a focus on improving physical function, fatigue, and quality of life upon completion. The results will inform the future design of the program on a larger scale, with the potential for it to be continued beyond the pandemic, to overcome geographical barriers and to widen access for participation.

## 2. Materials and Methods

### 2.1. Participants

This was a prospective, cohort observational study of 139 patients, which formed part of our service evaluation. Complete results were returned by 66 patients (34 males and 32 females); only patients with complete data were included in the final analysis of this study. From the end of March 2020, following the announcement of a UK national lockdown, we were able to adapt our traditional face-to-face service into a home-based program. Our study period was from April to December 2020. Eligible patients awaiting cancer treatment were identified from multiple centers and referred to a dedicated regional prehabilitation team providing home-based prehabilitation. 

### 2.2. Service Design

Our intervention was modelled on our existing in-person supervised prehabilitation program [4,5]. We were able to adopt a multi-modal telehealth-delivered home-based program. Our program consisted of the following four key interventions: (i) personalized training exercises, (ii) nutritional advice, (iii) medical optimization therapies, and (iv) psychological support. Patients were recruited from multiple hospital sites based in the South East of England. Patients were recruited to the study if they were (i) aged ≥18 years old, (ii) with a cancer diagnosis, and were (iii) due to undergo treatment. Patients had to have sufficient knowledge of English to understand and answer the questionnaires. The patients opted in or out of the prehabilitation service being offered. The patients consented to participating in the study and completing the questionnaires to be used in the analysis of this study. 

### 2.3. Referral Process

Several referral methods were made available. Healthcare professionals could either refer patients using an online form that was made available on the webpage of the service or an offline form, which could be later emailed to the prehabilitation team. To suit their convenience, a copy of the referral form was also integrated into the clinical software (“InfoFlex”, Kent and Medway Cancer Collaborative, United Kingdom) used by cancer nurse specialists (CNSs). Furthermore, a self-referral online form was created to allow patients to enroll themselves on the service.

### 2.4. Format of Prehabilitation Sessions

The program started within one week of the prehab team receiving the referrals and was considered completed immediately before the patients’ operating date or upon finishing their non-surgical cancer treatment. Surgical patients performed prehabilitation up until one week before their surgery. Non-surgical patients performed our interventional recommendations throughout their non-surgical cancer treatment. A physiologist conducted an initial screening interview with each patient either over the telephone or via video call. A baseline assessment was performed during this session. Information was obtained on the patient’s health status, past medical history (including smoking status and alcohol consumption), current medications, well-being, quality of life, and physical activity levels. The malnutrition universal screening tool (MUST) was used to determine whether the patients needed a clinical nutritional intervention [17]. Nutritional education and physical activity recommendations were provided and personalized according to the patient’s initial functional capacity. Following their initial contact, the patients were contacted up to twice a week based on their preferences and needs. The advice and recommendations provided during the initial screening were reinforced, and any questions or doubts were addressed during these sessions.

### 2.5. Intervention

#### 2.5.1. Physical Activity

Patients were recommended to perform moderate intensity physical activity (MPA) for at least 150 minutes per week [18]. The MPA was in the form of walking, cycling or any other activity that would increase their breathing frequency, whilst still being able to talk. Patients were also encouraged to perform home-based resistance exercises at least twice a week. They were provided with an online exercise video and/or exercise guides with a description of each exercise and advice on how to perform the exercises safely. These guides included six multi-joint resistance training exercises targeting all of the major muscle groups and were presented in a circuit format (sit-to-stand, wall-press, calf raises, unilateral row, knee extension, and shoulder press). Patients were encouraged to perform the circuit three times per session, for 8–10 repetitions per exercise per set. Further advice on exercise adjustments were provided to increase, or reduce, their difficulty. Patients waiting for colorectal, or urology operations were encouraged to perform pelvic floor exercises and those awaiting lung or breast surgery were encouraged to perform inspiratory muscle training exercises.

#### 2.5.2. Nutritional Education

Patients were encouraged to prioritize non-processed and minimally processed foods, whilst avoiding processed and ultra-processed foods. For these purposes, we referenced the NOVA guidelines, a food classification based on the extent and purpose of industrial food processing [19]. Furthermore, patients were also taught the importance of protein amounts and quality, whilst being recommended to aim for a minimum daily protein intake of 1.5 g/kg of ideal body weight [20]. To facilitate this, patients were provided with a list of foods highlighting the amounts of protein contained in various serving sizes. Patients with a low MUST score at baseline assessment were advised to consume supplemental nutritional shakes [21].

#### 2.5.3. Medical Optimization Therapies

Smokers at baseline assessment were offered nicotine-replacement therapies and help to quit via smoking cessation services, and those who reported drinking more than the maximum alcohol intake recommended by the UK Chief Medical Officers’ Alcohol Guidelines were offered advice via alcohol moderation services [22]. If they declined, they were reminded that this support could be accessed at any time.

#### 2.5.4. Psychological Support

Patients were offered individual counselling sessions. If the patient accepted this intervention, each patient had at least one session with an MBACP Accredited counsellor, who assessed them and determined the nature and intensity of the intervention required. If the patient declined, they would be reminded that they could request to access this support at any time.

### 2.6. Signposting to Wellbeing Navigation Services

If, during the initial screening or the subsequent prehabilitation sessions, patients were found to need any additional support with aspects not covered in the prehabilitation program, they were offered a referral to wellbeing navigation services. These services offer a personalized approach, enabling people to remain in their own home, increase their ability to take control and self-manage, and can help people to access a wide range of services, including, but not limited to, befriending, home safety, financial support, housing, domestic support, shopping, and transport.

### 2.7. Outcome Measures

The primary outcome was to evaluate the feasibility of the service. Feasibility was assessed using recruitment rate, retention rate, and patient experience. The recruitment rate was determined by the number of eligible patients who were referred to the service, compared to the number of patients that were enrolled. Reasons for non-participation were also recorded. Retention rate was defined as the number of patients who were able to complete the program up until the week of their operation, and/or upon completion of their non-surgical cancer treatment, out of the number of patients enrolled. We undertook a focus group session with participating patients to obtain patient feedback on their experiences at the end of the study period.

The secondary outcome was to assess any changes in patient-reported outcome measures upon completion of prehab. We sought to evaluate the reduction in symptom burden, particularly on health-related quality of life and cancer-related fatigue. For this reason, we selected two questionnaires, the EQ-5D-3L and the Functional Assessment of Chronic Illness Therapy (FACIT)-Fatigue Scale. Patients completed these questionnaires twice [23,24,25]. The first set of questionnaires was completed upon enrolment to the program, which formed the baseline assessment. These same questionnaires were repeated upon completion of the program. For the surgical patients, the questionnaires were completed in the 24–48 h prior to surgery and for the non-surgical patients, these were completed the week after finishing their cancer treatment.

The EQ-5D-3L is a tool for measuring health-related quality of life. The EQ-5D-3L has been developed as a standardized validated clinical outcome measurement and has been chosen by the Department of Health and Social Care in England as a quality indicator to evaluate the provision of patient care [26]. The EQ-5D-3L descriptive system comprises the following 5 dimensions: mobility, self-care, usual activities, pain/discomfort, and anxiety/depression. Each dimension has the following three levels: no problems (coded as 1), some problems (coded as 2) or extreme problems (coded as 3). The EQ-5D-3L describes 243 unique health profiles (3^5^). These health profiles can be transformed into values by using the pertinent EQ-5D-3L value set. For this purpose, the UK EQ-5D-3L value set was utilized. The EQ visual analogue scale (VAS) records the patient’s self-rated health on a vertical VAS, where the end points are labelled “The best health you can imagine” (equivalent to 100%) and “The worst health you can imagine” (equivalent to 0%).

The FACIT-Fatigue Scale is a validated tool to measure fatigue amongst patients. The questionnaire was developed initially to evaluate cancer-related fatigue and was, therefore, deemed the most appropriate for our cohort of patients. The FACIT-Fatigue Scale is a short, 13-item, easy-to-administer tool used to measure an individual’s level of fatigue during their usual daily activities over the last 7 days. The level of fatigue is measured on a 5-point Likert scale (from 4 = not at all fatigued to 0 = very much fatigued). The scores from this scale can range from 0 to 52, and a higher score represents less fatigue. The EQ-5D-3L and the FACIT-Fatigue Scale were chosen for their ease of application, reliability, reproducibility, and validity.

### 2.8. Statistical Analysis

The responses from the EQ-5D-3L and the FACIT-Fatigue Scale were compared in patients who enrolled in prehabilitation, upon starting the program and immediately before undergoing surgery, to determine the changes derived from the prehabilitation program. Pre- and post-prehabilitation EQ-5D-3L scores were compared by calculating the number of patients per dimension and the levels of problems. Pre- and post-prehabilitation EQ-VAS, FACIT-Fatigue Scale and EQ-5D values were compared by using the Wilcoxon signed-rank test. A *p* value of <0.05 was considered statistically significant. Statistical analysis was performed using SPSS software and Windows Microsoft Excel.

### 2.9. Ethical Considerations

Our service innovation was registered as an Innovation and Service adaptation with the respective local Audit/Quality Improvement centers by clinicians in those centers. Approval was granted by each local department. Eligible patients were identified and approached about the study following confirmed cancer diagnosis. Eligible patients who agreed to participate in prehabilitation provided informed consent for their results to be used in the analysis of this study. Data is collected routinely, as part of our service offering, which is in partnership with the Public Health Team within our local council. Data collection and evaluation has been approved by Public Health.

## 3. Results

### 3.1. Feasibility

#### 3.1.1. Recruitment Rate

**Figure 1:**
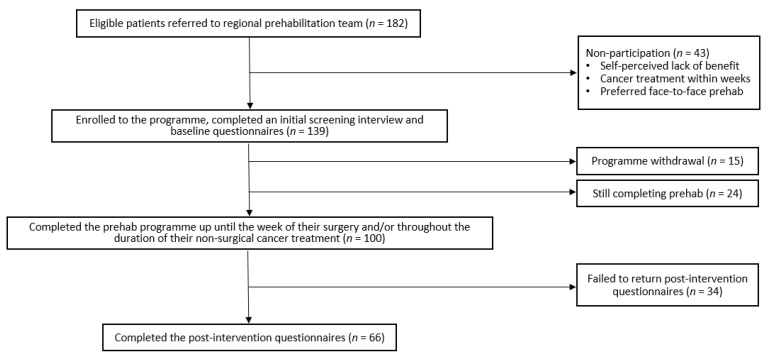
Patient flow chart.

The patient flow chart is presented in Figure 1. A total of 182 patients were referred to our service between late March and December 2020. The patients were referred from nine hospital trusts and three general practices. These sites covered a wide geographical area across the south of England, including London, Kent, Surrey, Oxfordshire, and Hertfordshire. The patients were referred from multiple cancer specialties, including colorectal (*n* = 102), urology (*n* = 42), breast (*n* = 18), lung (*n* = 13), hepatobiliary (*n* = 2), neurosurgery (*n* = 2), and unknown (*n* = 3, the patients did not disclose). The referral sources varied; cancer specialist nurses (*n* = 89), clinical support workers (*n* = 30), hospital doctors (*n* = 35, including anesthetists, oncologists, surgeons), patient self-referrals (*n* = 11), general practitioners (*n* = 2), physiotherapists (*n* = 1), and unknown (*n* = 14, the referrers did not disclose). Of the 182 patients that were referred, 139 (76%) patients agreed to partake, and 43 (24%) patients declined or were unable to participate. The most common reasons for non-participation included a self-perceived lack of benefit (e.g., they felt they were already fit and healthy, or had existing multiple co-morbidities and felt the program was not suitable for them), their cancer treatment was due within weeks, and they did not want to enroll unless it was face-to-face.

#### 3.1.2. Retention Rate

Amongst the 139 patients that were enrolled onto the program, 100 (72%) patients were able to adhere to the interventional recommendations up until the date of their cancer treatment. At the time of writing this study, 24 (17%) patients were still completing prehabilitation and 15 (11%) patients had withdrawn from the program. Due to the project being continuous, a cut-off point for early analysis had to be selected. The initial point of review would take place when 100 patients had completed the program. The most common reasons for program discontinuation included patients feeling “overwhelmed”, either by the number of hospital appointments required for scans and/or doctor visits, the amount of information being provided or from their cancer diagnosis itself.

### 3.2. Patient Characteristics

Among the 100 patients that completed the program, only 66 patients returned completed questionnaires. The patients that did not return full results were excluded from the analysis of the study. Before and after prehabilitation, data were obtained from the 66 patients (age (median (interquartile range)): 67 (60–73) years, BMI: 27.9 (24.0–31.2) kg·m^−2^). The prehabilitation program had a mean duration of 4 (3–9) weeks. The patients received five (4–8) telephone/video calls during this time. The length of stay (3 (1–6) days) could be collected for the 53 patients who underwent surgery. From those patients, a 90-day readmission could be obtained for 44, of whom 10 needed readmissions. Table 1 provides a summary of the characteristics of those patients’ who completed the program and returned results.

### 3.3. Patient-Reported Outcomes

**Figure 2:**
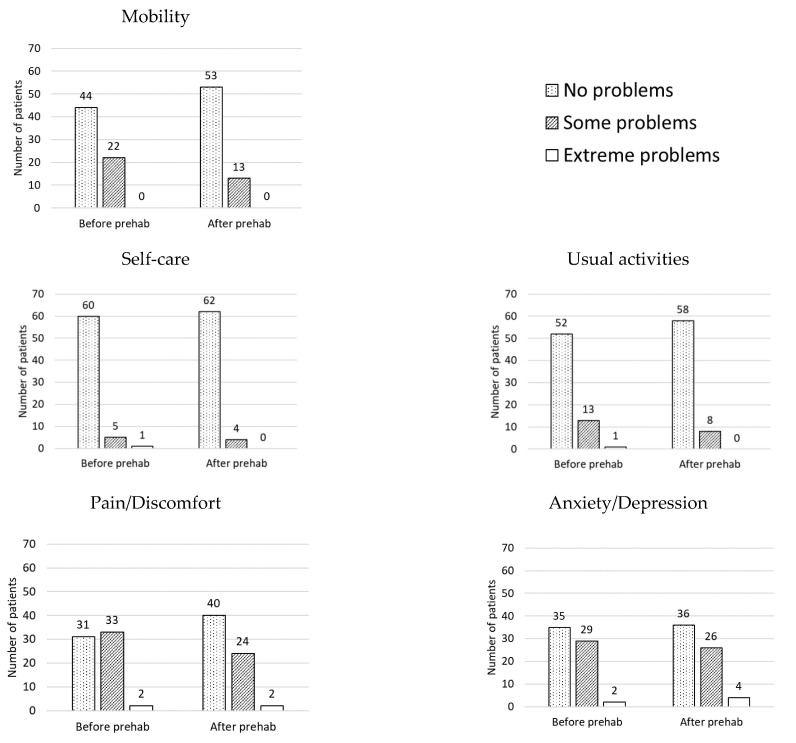
Number of patients per dimension (mobility, self-care, usual activities, pain/discomfort, and anxiety/depression) and level of problems (no problems, some problems, extreme problems), before and after undergoing prehabilitation. A greater number of patients selecting “No problems” in the different dimensions after undergoing prehabilitation suggests the intervention was effective in improving quality of life.

**Figure 3:**
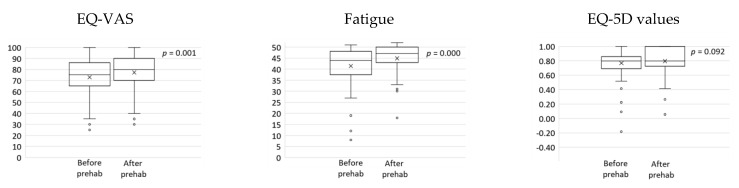
Boxplots depicting self-rated health (EQ-VAS), fatigue, and EQ-5D values, before and after prehabilitation. Statistically significant improvements were found in self-rated health (*p* = 0.001) and fatigue (*p* = 0.000).

The results of the changes after home-based prehabilitation are provided in Figure 2 and Figure 3 below. The EQ-5D-3L analysis showed an increased number of patients in the “No problems” category for all of the dimensions after completing prehabilitation. In addition, a greater number of patients scored “Extreme problems” in the dimension “Anxiety/Depression” after completing prehabilitation. These changes are represented in Figure 2. There were statistically significant improvements in the EQ VAS (median (interquartile range); before: 75 (65–86) vs. after: 80 (70–90); *p* = 0.001) and the FACIT-Fatigue Scale (before: 44 (38–48) vs. after: 47 (43–50); *p* = 0.000). There were no statistically significant changes in the EQ-5D values (before: 0.796 (0.691–0.857) vs. after: 0.796 (0.725–1.000); *p* = 0.092). Figure 3 demonstrates boxplots for the variables before and after prehabilitation.

### 3.4. Patient Feedback

#### 3.4.1. Benefits

We conducted a focus group with seven patients who had completed prehabilitation. The patient feedback had been positive overall. The patients welcomed the telehealth-delivered format, citing flexibility, accessibility, social support, and preventing the need to exercise in front of others as perceived benefits. A home-based program mitigated the need to travel to the prehabilitation unit, which provided much desired flexibility around medical and/or personal commitments. In addition, we were able to widen access to those who may have not been able to attend the face-to-face in-hospital sessions (pre-COVID era), because of the unavailability of hospital transport or they were otherwise too unwell to drive. Notably, we were able to overcome geographical barriers; patients living outside of the region were able to access the service when they might not have been able to due to the distance and time it takes to travel to our dedicated centers. The patients appreciated the social support from our team during the periods of lockdown. The patients were able to maintain uninterrupted support from the prehab team due to our program adaptation, whilst some other services were paused. One patient described the fear of embarrassment of having to exercise in front of others and welcomed performing exercises at home as a safe alternative to face-to-face exercise sessions.

#### 3.4.2. Challenges

Patients cited several challenges associated with a home-based program, which were centered around the use of digital devices and not being able to meet other people. A few patients cited a self-perceived lack of digital ability and literacy. Despite there being a paucity of digital confidence, participants were willing to engage in the service, and saw the program as an opportunity to upskill. Therefore, digital literacy did not become a significant barrier to participation, as anticipated. However, the patients in the focus group recognized digital resources may be cost prohibitive for some patients. The absence of group sessions meant that there was no opportunity for peer support, which they felt was well placed alongside exercise, diet, and medical recommendations. The patients felt that it would be beneficial to meet and engage with people in a similar position to provide support throughout their cancer treatment journey and beyond. In addition, they felt that home-based approaches relied further on self-motivation compared to attending face-to-face appointments.

## 4. Discussion

The coronavirus COVID-19 pandemic has impacted the delivery of healthcare services around the world. To reduce the risk of exposure to coronavirus for our patients, we have had to limit non-essential hospital visits by restricting face-to-face prehab. Consequently, we have had to adapt and innovate our prehabilitation program accordingly into a “virtual” home-based format. This study investigated the feasibility of a telehealth-delivered home-based prehabilitation program for cancer patients prior to surgery and/or during non-surgical cancer treatment, and the effects on patient-reported outcomes. Our results showed that home-based prehabilitation is a feasible intervention. Our telehealth-delivered adaptation has been welcomed by both patients and healthcare professionals within our community. There was a high acceptance rate (76%), a high adherence rate (72%), and positive patient feedback.

There are several reasons to account for our feasibility outcomes. We have been able to improve on some of the recruitment problems highlighted in previous studies [5,27]. By offering a home-based format, we have been able to address key concerns relating to hospital transport, in-person supervised session availability and timings (e.g., availability of exercise equipment and space), and the need to accommodate our interventional recommendations around medical and/or personal commitments. Furthermore, the telehealth approach to delivering prehabilitation has allowed our program to expand its reach beyond geographical barriers. We have been able to offer the intervention across the entire county, again addressing accessibility, and the elements of post code lottery, which is often cited as a reason for healthcare inequality [28]. In addition, telehealth-delivered home-based prehabilitation may be a cost-effective approach to delivering prehab that is not bound by locality, as we have a specialist centralized hub, with a dedicated team. By providing a centralized system, we can overcome geographical barriers and allow patients across the region accessibility. Beyond the pandemic, home-based prehabilitation should be continued to widen participation.

Prehabilitation involves health-optimizing interventions and has been shown to enhance health and treatment outcomes. In the setting of the COVID-19 pandemic, prehabilitation may counteract the unintended consequences of shielding, that may result in physical deconditioning and immobility [29,30,31]. Our program involved regular “check-ins” with our patients and a multi-modal approach to prehabilitation. We have demonstrated that our intervention can contribute to important changes in global health and fatigue. Our interventions resulted in improvements in both of the questionnaires used to assess patient-reported outcomes (the EQ-5D-3L and the FACIT-Fatigue Scale). There were statistically significant improvements in the EQ VAS and the FACIT-Fatigue Scale, which assesses self-perceived health and fatigue, respectively. There were no statistically significant changes in the EQ-5D values, likely due to the patients being in reasonably good health at baseline, despite their cancer diagnosis. Furthermore, we observed a translocation of patients towards the “No problems” level in every dimension of the EQ-5D-3L, which reinforces the idea that our intervention had a measurable effect in our cohort of patients. Interestingly, we also observed that more patients scored “Extreme problems” in the “Anxiety/Depression” dimension after finishing the program (before: *n* = 2 vs. after: *n* = 4). This result could be owed to 59 (89%) patients from the cohort having surgery after completing prehabilitation. Having surgery in the following 24–48 h could, indeed, explain the increased levels of anxiety.

### Limitations

There are several limitations associated with our study. The patients were heterogenous in terms of cancer origins, treatment, surgery, and duration of prehabilitation. The patients selected to participate in the program themselves, resulting in a self-selection bias. We did not have a control group for comparison. Without a control group, we are not able to say whether our program alone would have improved their outcomes. We had a small sample size (only 66 patients returned results and were included in the final analysis). Subsequently, the narrow population limits the interpretation of our findings. Further studies involving a larger cohort of patients and a control group (consisting of the patients who declined to participate) are required to support the generalization of our conclusion. Furthermore, we did not have functional assessments, as this was limited by equipment, given that patients were performing this program at home and not at our dedicated center. However, we did encourage our patients to follow the recommendations prescribed to them, but we could not ascertain their adherence to these.

The aim of our project was to adapt and implement a home-based prehabilitation service, with a focus of providing a solution to mitigate the consequences of shielding during the pandemic. We collected data when possible, but due to the prioritization of our service delivery, some circumstances made data collection more challenging than others. For instance, a lack of time during consultations; patients did not feel like going through the questionnaires; patients having hearing impairments, requiring the session to be interpreted through their partner/relative; patients not knowing how to answer the questionnaires; and patients completing the questionnaires incorrectly (i.e., choosing two options when the questions ask for one).

## 5. Conclusions

This study demonstrated the feasibility and effects of a telehealth-delivered home-based prehabilitation program for cancer patients prior to undergoing surgery and/or during non-surgical treatment. There was a high recruitment rate (76%), a high retention rate (72%), and positive patient feedback. The patient-reported outcomes improved post-intervention. The patients significantly improved their self-perceived health and fatigue. Our program is a feasible and effective intervention to mitigate the consequences of shielding during the pandemic.

## Figures and Tables

**Table 1:** Summary of patients’ characteristics.

Patients’ Characteristics	*N*	%
Gender		
Female	32	48
Male	34	52
Ethnicity		
Asian or Asian British: Any other Asian background	1	2
Black, African, Caribbean, or Black British: Caribbean	2	3
White: Any other White background	2	3
White: English, Welsh, Scottish, Northern Irish or British	61	92
Specialty		
Colorectal	41	62
Breast	7	11
Lung	3	4
Urology	15	23
Treatment		
Chemo and/or radiotherapy	7	11
Surgery	54	82
Chemo and surgery	5	7
Duration of prehabilitation		
<2 weeks	5	8
2–4 weeks	27	41
4–6 weeks	12	18
6–8 weeks	3	5
≥8 weeks	19	29
Counselling		
Accepted	13	20
Declined	53	80
Alcohol intake		
≤14 units per week	52	79
>14 units per week	14	21
Alcohol moderation referral		
Accepted	0	0
Declined	14 *	100
Smoker		
No	60	91
Yes	6	9
Smoking cessation referral		
Accepted	2	33
Declined	4	67
High-risk of malnutrition		
Yes	3	5
No	63	95

* Of the 14 patients drinking >14 units of alcohol per week, 2 were already receiving support.

## Data Availability

The data presented in this study are available on request from the corresponding author. Data is not publicly available due to privacy concerns.
